# The Dead

**Published:** 1891-01-03

**Authors:** 


### THE DEAD.

Space warns us to be brief in our further references to public events. But we cannot forbear calling to recollection the loss which the public and the medical profession sustained during the year by the death of Dr. Matthews Duncan and several other well-known medical practitioners. Dr. Matthews Duncan was hardly an aged man, as medical men are called aged. Both as a physician and as a man he was earnest, honest, and capable; and he had his reward in the confidence of his brethren and the admiring affection of a large and discriminating section of the public. Towards the close of the year also died Lady Rosebery, still comparatively in her youth. She was a doer of good deeds and a friend of those that did them ; she was officially connected with the National Pension Fund for Nurses, and a warm supporter of hospitals and deserving charitable institutions. The world of charity is made poorer by Lady Rosebery's death, and all good people who knew her mourn her loss.

